# Mechanistic understanding of heat stress in cattle reproduction toward pharmacological strategies: review

**DOI:** 10.5713/ab.250531

**Published:** 2025-10-28

**Authors:** Ismail Shaleh, Sung Woo Kim, Tae Sub Park, Joonghoon Park

**Affiliations:** 1 Graduate School of Int’l Agriculture Technology, Green Bio Science & Technology, Seoul National University, Pyeongchang, Korea; 2 Department of Biology, Faculty of Mathematics and Natural Science, IPB University, Bogor, Indonesia; 3 Hanwoo Research Institute, National Institute of Animal Science, Rural Development Administration, Pyeongchang, Korea; 4 Department of Agricultural Biotechnology, Research Institute of Agriculture and Life Science, Seoul National University, Seoul, Korea

**Keywords:** Cattle, Heat Stress, Mechanism, Pharmacology, Reproduction

## Abstract

Heat stress is a major environmental challenge that compromises reproductive performance in cattle, particularly under the intensifying conditions of global climate change. This review provides a comprehensive overview of how heat stress impairs bovine reproduction at the physiological, cellular, and molecular levels and explores practical strategies for mitigation. In females, heat stress disrupts hormonal regulation, estrous behavior, and ovulation, and diminishes oocyte and embryo quality, resulting in reduced conception rates and increased pregnancy losses. In males, prolonged heat exposure impairs spermatogenesis and semen quality and delays post-stress recovery. Dairy cattle are especially vulnerable due to the elevated metabolic demands of lactation. Although beef cattle exhibit greater thermotolerance, they also experience reduced fertility under prolonged heat stress, particularly during breeding and early gestation. At the cellular level, heat stress triggers oxidative damage, mitochondrial dysfunction, immune dysregulation, and altered epigenetic and cytoskeletal dynamics. Integrative transcriptomic analyses across key reproductive tissues reveal both conserved and cell type–specific molecular responses. These include activation of inflammatory and apoptotic pathways, suppression of chaperone-mediated protein folding and hormone receptor signaling, and downregulation of uterine receptivity programs. To counter these effects, current strategies involve environmental modifications, genetic selection for thermotolerance, and supportive treatments such as antioxidants, methyl donors, and hormonal protocols. However, most interventions remain symptomatic and nonspecific. Future efforts must prioritize mechanistically grounded approaches that target the molecular drivers of heat-induced reproductive dysfunction. Continued research integrating multi-omics, network-based modeling, and pharmacological discovery will be critical to developing next-generation solutions that enhance reproductive resilience and sustainability in cattle production systems.

## INTRODUCTION

The rise in global temperatures has led to more frequent and intense heat stress episodes, adversely impacting fertility and production in both dairy and beef industries. During periods when the temperature-humidity index (THI) exceeds 72, cattle struggle to maintain effective thermoregulation [1]. This disruption in thermoregulation leads to reduced conception rates, compromised fetal development, and economic losses. Therefore, it is essential to understand how these environmental conditions interfere with reproductive processes. Research on the reproductive impact of heat stress dates back to the 1980s, when early studies established its link to reduced pregnancy rates and disrupted estrous cycles. Cattle exposed to THI above 72 exhibited prolonged inter-estrus intervals and weaker estrous behavior, leading to lower mating success compared to thermoneutral conditions [2]. These disruptions were attributed to heat-induced hormonal imbalances, such as elevated progesterone and inappropriately reduced preovulatory estradiol [3]. Since then, advances in genetics, molecular biology, and environmental management have deepened our understanding of these mechanisms. Recent studies highlight how heat stress induces cellular stress responses, including increased reactive oxygen species (ROS) production and oxidative damage in reproductive cells. Climate change projections underscore the urgency of addressing heat stress in cattle reproduction. According to Intergovernmental Panel on Climate Change (IPCC) reports ( https://www.cigionline.org/), global surface temperatures are expected to rise by 1.5°C–2.0°C by mid-century, with more frequent and prolonged heat waves in major livestock-producing regions. Predictive models indicate that the number of days exceeding a THI of 72, the threshold for heat stress in cattle, may double in subtropical areas by 2050. Such projections highlight the increasing vulnerability of cattle reproduction to environmental stressors and emphasize the critical need to develop sustainable mitigation strategies [4]. In this review, we examine the physiological and molecular mechanisms underlying heat stress-induced reproductive impairments, including disruptions in hormonal balance, embryonic development, and cellular responses in the reproductive tract. Lastly, we explore mitigation strategies, from genetic selection for heat tolerance to supportive treatments, and discuss emerging approaches to reduce the negative impact of heat stress on cattle reproduction.

## IMPACT OF HEAT STRESS

### Physiological changes

When ambient temperatures exceed the thermoneutral range, rectal temperatures often rise above 39°C, which can reduce fertility and milk production. Cattle cope by increasing respiration and peripheral blood flow to release excess heat, with respiration rising from about 30 to 50 breaths per minute in comfortable conditions to over 80 or even 100 under severe heat stress. Heart rate also increases from around 70–80 to over 100 beats per minute under extreme conditions. This trend appears consistently in both natural and controlled environments. Under mild conditions, body temperature, respiration, and heart rate remain stable within normal ranges, while hotter conditions lead to higher rectal and surface temperatures and a marked increase in respiration. Cooling systems help moderate these responses, although core temperature and respiration still rise when heat load is high (Table 1) [5,6].

Heat stress also reduces feed intake by about 30% to 35% as an adaptive response that lowers internal heat from digestion and fermentation, but this subsequently decreases energy available for milk synthesis [5]. Combined with direct suppression of mammary metabolism, this leads to 10%–20% declines in milk yield, depending on severity and duration. When the THI exceeds 68–72, dry matter intake often drops by nearly a third, accounting for about half of the reduction in milk production, with the remainder caused by altered nutrient partitioning and disrupted metabolism [7]. Heat-stressed cattle enter a state of negative energy balance due to reduced feed intake, which normally triggers mobilization of non-esterified fatty acids (NEFA) from fat stores. Reported plasma NEFA concentrations under heat stress range from 139 to 305 μEq/L, whereas pair-fed cows exhibit approximately a twofold increase reaching 506 μEq/L, indicating that reduced intake is the primary driver of this response [6].

Dairy cattle face a much higher internal heat load due to lactation. For example, cows producing 31.6 kg of milk daily generate about 1.5 times more metabolic heat than non-lactating cows [8]. Consequently, their respiratory rate can increase to 80–120 breaths per minute with oxygen consumption around 5.5 L/min, while beef cattle show only moderate increases (20–40 breaths/min, 3.5 L/min) [9,10]. Holsteins, in particular, struggle with heat dissipation because of their large metabolic output and small surface-to-mass ratio. In contrast, beef breeds such as Brahman possess traits that enhance thermoregulation, including short thin coats, larger surface area, and more active sweat glands. Histological features such as reduced epidermal thickness and larger, shallower sweat glands further support cooling [11,12]. Genetic factors also contribute: beef breeds often show stronger HSP70 expression [13], and the SLICK1 mutation in PRLR, introduced into some dairy breeds, reduces insulation and improves thermotolerance without majorly compromising milk yield.

### Reproductive dysfunction

Heat stress disrupts the endocrine axis in reproduction. Preovulatory estradiol decreases to 4–5 pg/mL at 32°C compared to 8–10 pg/mL at 21°C, weakening estrous expression [14]. Normally, estradiol rises sharply before the LH surge, but in heat-stressed cattle the peak remains lower, with only 37% of heifers showing a surge versus 87% under thermoneutral conditions. Peak LH concentrations also fall from 61 ng/mL to 45 ng/mL [15,16]. Progesterone dynamics are disturbed as well, often rising up to 9 ng/mL during acute stress compared to 5 ng/mL [16], and in hotter months luteal peaks are lower and decline more steeply [17]. These endocrine changes translate into impaired reproductive outcomes in females. Weak estrous behavior complicates detection and timing of AI [14,18]. Conception rates drop from 30.43% at THI 55 to 25.47% at 70, and to 18.97% at 77 [19]. Heat stress also impairs ovulation, reduces endometrial receptivity, and creates suboptimal conditions for embryo implantation and survival. Early embryonic development is particularly vulnerable to elevated temperatures due to reduced uterine blood flow and increased oxidative stress, with embryonic mortality rising from 2% to up to 12%, especially between days 34–90 of gestation [20]. Heat exposure during late gestation shortens the average gestation length by about 3.2 days, which partially explains the reduced birth weight by limiting time for fetal growth. However, heat stress also directly affects fetal development through impaired placental function, reduced nutrient and oxygen transfer, and altered metabolic and hormonal environments [21,22]. Similar mechanisms have been described in heat-stressed sheep, including reduced uterine and umbilical blood flow, impaired placental nutrient transport, and fetal hypoxia [23,24]. As a result, calves born to heat-stressed dams show about 12.4% lower birth weights and 10.4% lower weaning weights, underscoring the importance of minimizing heat stress during pregnancy to improve offspring survival and growth [20].

Male fertility is also affected, though less dramatically. Heat exposure disrupts spermatogenesis, leading to DNA fragmentation, mitochondrial dysfunction, and lipid peroxidation. Progressive motility declines by 30%–40%, viability by up to 50%, and morphological abnormalities increase from 5% to 20% after stress [25]. These effects are particularly pronounced 14–21 days post-heat stress, coinciding with the maturation period of spermatids. Recovery of semen quality requires 6–8 weeks post-stress, aligning with the full spermatogenic cycle [25,26]. Reduced testosterone synthesis further contributes to lower sperm concentration and ejaculate volume. The economic consequences are substantial. In dairy cattle, conception rates fall from 52% under thermoneutral conditions to 32% during heat stress, contributing to estimated annual losses of $1.2 billion in the U.S. [19,27]. Beef cattle are more resilient due to lower metabolic burden, but still experience 10%–15% declines in conception, 15%–20% reductions in calf crop in hot regions, and 2–5 kg lower calf birth weights [28,29]. These outcomes demonstrate that while dairy cattle are particularly vulnerable, both sectors face significant productivity losses linked to heat-related reproductive dysfunction.

## MOLECULAR AND CELLULAR LEVEL CHANGES

### Oocyte

Heat stress disrupts nuclear-cytoplasmic maturation in oocytes and impairs mitochondrial function, which is essential for ATP production required for fertilization and early embryonic development. Exposure of bovine oocytes to 39.5°C–40.5°C for 6 to 24 hours significantly reduces maturation rates, declining from about 86% in controls to 36% after prolonged treatment [30]. Studies have also shown that oocytes subjected to heat stress around 41°C exhibit a marked decline in mitochondrial membrane potential, leading to diminished energy reserves, meiotic arrest, and increased cellular organelle stress [31]. Additionally, heat stress induces excessive ROS production, resulting in oxidative damage that compromises oocyte developmental potential. This oxidative imbalance has been directly linked to lower cleavage and blastocyst formation rates, ultimately reducing the reproductive capacity of heat-stressed oocytes [32]. Heat stress also significantly alters the fatty acid composition of oocytes, increasing the proportion of saturated fatty acids such as stearic acid (C18:0) and palmitic acid (C16:0). This shift can reduce membrane fluidity and impair developmental competence after fertilization [33]. Conversely, oocytes collected during winter exhibit higher levels of unsaturated fatty acids, which may enhance membrane stability and improve oocyte developmental potential [33]. These compositional differences may partly explain seasonal fertility variations, highlighting the beneficial effects of winter-derived fatty acid profiles on oocyte quality and developmental success.

### Granulosa cell

Heat stress induces apoptosis in bovine granulosa cells primarily through mitochondrial disruption and oxidative stress. Elevated ROS levels promote apoptosis by both activating the caspase cascade and upregulating pro-apoptotic gene expression via nuclear translocation of forkhead box (FoxO) transcription factors [34]. Consistent with these mechanisms, heat-stressed granulosa cells exhibit increased oxidative stress, elevated HSP70 and HSP90 levels, decreased viability, and enhanced early apoptosis [35]. Although the induction of HSPs represents a transient protective response, it appears to be insufficient to counteract the cumulative pro-apoptotic signals. Under thermoneutral conditions, the phosphatidylinositol-3 kinase (PI3K)/Akt signaling pathway supports cell survival by inhibiting pro-apoptotic factors [36]. However, heat stress suppresses the activity of the PI3K/Akt pathway [37], thereby weakening its protective effect and further exacerbating apoptotic cell death. This disruption ultimately contributes to granulosa cell loss and impaired follicular development. Beyond cell survival, heat stress also impairs granulosa cell endocrine function. Specifically, granulosa cells produce significantly less estradiol at 40.5°C than at the thermoneutral temperature, regardless of the season when the cells were collected [38]. This estradiol reduction disrupts the maternal estrous cycle and further compromises reproductive performance.

### Embryo

Heat stress during early embryonic development significantly impairs developmental competence in bovine embryos. Exposure to 41°C leads to a reduction in blastocyst formation by up to 40%, primarily due to cytoskeletal disruption, organelle redistribution, and mitochondrial swelling [39]. These changes impair ATP production and increase ROS levels [32], which in turn promote apoptosis and reduce cellular viability [40]. Experimental observations confirm that oocytes and early-stage embryos are highly vulnerable to elevated temperatures. Under heat stress, the proportion of oocytes developing to the blastocyst stage significantly decreases from 42.3% to 23.5%. The two-cell stage is particularly sensitive, showing a dramatic decline in developmental competence from 26.5% to 0.1%, while the four- to eight-cell stage also exhibits a marked reduction from 24.5% to 10.3%. In contrast, compacted morulae maintain similar developmental rates under heat stress compared to thermoneutral conditions. Additionally, heat stress substantially increases the incidence of retarded or morphologically abnormal embryos, with rates rising from 29% to 70% [41,42]. These findings underscore the critical importance of temperature regulation during the early stages of embryonic development to preserve viability and ensure successful progression to the blastocyst stage. Heat stress in preimplantation bovine embryos also disrupts epigenetic regulation by inducing hypomethylation of imprinted genes such as *H19* and insulin-like growth factor 2 receptor (*Igf2r*), both of which play critical roles in early development [43,44]. These alterations particularly affect the normally methylated paternal alleles, leading to loss of imprinting and aberrant gene expression that may impair embryonic growth and developmental competence [44]. Additionally, heat stress often leads to global DNA hypomethylation and histone H3K9 hypoacetylation, reducing expression of antioxidant genes like SOD2 (*MnSOD*), which weakens the embryonic defense against ROS, further hindering development [45].

### Oviduct

The oviduct also suffers from heat-induced alterations, which affect the microenvironment essential for sperm transport, fertilization, and early embryonic support. In heat-stressed cattle, oviductal secretions decrease significantly, affecting key components necessary for sperm motility and capacitation [46]. For instance, the concentrations of glycoproteins such as oviductin and albumin are notably reduced under thermal stress [46,47]. Oviductin facilitates sperm-oocyte binding and enhances the acrosomal reaction, both of which essential for sperm penetration to the oocyte [48], while albumin assists in cholesterol efflux, a critical step in capacitation [49]. Quantitatively, studies show that oviductal protein concentrations can drop by approximately 0.2 to 0.3 mg/mL under heat stress conditions, impacting the support required for successful fertilization [46]. Additionally, heat stress affects the oxidative balance within the oviduct, increasing ROS levels by 2 to 3-fold [50] that further impair the ability of the oocyte to undergo successful fertilization and hinder the initial stages of embryonic development.

### Uterus

The uterus is highly sensitive to heat stress and elevated ambient temperatures increase uterine tissue temperature by approximately 0.5°C [51]. This increase restricts uterine blood flow, reducing oxygen and nutrient availability, which are essential for embryo survival and implantation [17,52]. One of the key hormonal changes in heat-stressed cattle is the elevation of prostaglandin F2-alpha (PGF2α) which is associated with luteolysis [53]. Under heat stress, PGF2α concentrations can exceed 250 pg/mL, shortening the luteal phase and significantly increasing the risk of early embryonic loss [53]. Heat stress also disrupts the balance of cytokines and growth factors essential for uterine receptivity. Insulin-like growth factor-1 (IGF-1), normally present at around 100 ng/mL, plays a key role in cell survival and development but is suppressed under heat stress reducing its thermoprotective effects [54]. Conversely, tumor necrosis factor-alpha (TNF-α) levels can rise above 10 pg/mL, triggering an inflammatory response detrimental to embryo attachment [55]. Furthermore, epidermal growth factor (EGF), essential for cellular proliferation and uterine remodeling, drops below the optimal range of 4.7–13.5 ng/mL, limiting the structural support required for successful implantation [56]. These hormonal and inflammatory disruptions create oxidative stress within the uterus, significantly reducing endometrial receptivity and implantation success [57].

### Sperm

Heat stress disrupts spermatogenesis by impairing mitochondrial function and increasing ROS, leading to oxidative damage in sperm DNA, lipids, and membranes [58]. These effects compromise both sperm morphology and function, ultimately reducing fertilization capacity [59]. In bulls exposed to prolonged temperatures above 35°C, sperm motility decreases by approximately 25% [60]. Additionally, the proportion of morphologically abnormal sperm such as those with deformed heads, midpiece defects, and coiled tails increases from 5% to 20% under heat stress conditions [61]. Under controlled experimental conditions, testicular heat stress caused a progressive decline in sperm motility from day 14 and a significant increase in morphological abnormalities, which began to rise as early as day 7. Membrane integrity was compromised, with plasma membrane damage appearing from day 14 and acrosomal membrane disruption persisting throughout the study. Mitochondrial membrane potential declined earlier, showing disruption by day 7 and remaining low thereafter. The proportion of functionally competent sperm, characterized by intact membranes and high mitochondrial activity, was markedly reduced, indicating cumulative and time-dependent deterioration in sperm structure and function [58]. Research indicates that oxidative damage to sperm membrane lipids increases nearly 2.5-fold under heat stress particularly in polyunsaturated fatty acids and contributes to membrane instability and acrosomal dysfunction [58,62]. Recovery from heat-induced spermatogenic damage is often prolonged, with full restoration of sperm quality and quantity taking up to 10 weeks after heat exposure ends [63]. This extended recovery period underscores the long-lasting effects of thermal stress on male fertility, affecting breeding efficiency and reproductive outcomes over successive cycles.

## MITIGATION STRATEGIES

### Environmental management

Managing heat stress in cattle requires well-designed facilities to regulate body temperature, focusing on shaded areas, ventilation, and water availability. Shade is fundamental, and studies indicate that shaded cattle maintain body and rectal temperatures to around 2°C lower than unshaded counterparts, translating to a 19.1% higher conception rate [64]. Ventilation systems, combining fans and sprinklers, enhance cooling through evaporation. Data from commercial dairy cattle in hot regions show that facilities with shade, fans, and sprinklers keep cattle rectal temperatures below 39°C [65], which correlates with conception rates as high as 50%, in contrast to only 10% conception in uncooled environments [66]. Ample water access is also crucial. High temperatures increase cattle water intake, and cool, accessible water stations support effective thermoregulation, which is vital for reproductive performance. Facilities that encourage drinking throughout the day, especially during peak heat, help cattle stabilize internal temperatures, supporting their overall health and reproduction. Additionally, feeding cattle during cooler phases of the day, such as early morning or evening, minimizes metabolic heat produced during digestion, thereby reducing internal heat stress [67]. Targeted cooling around insemination provides additional reproductive benefits. Studies indicate that short-term cooling for 2–3 days before and 5–6 days after breeding improves pregnancy rates, increasing conception rates from 6.2% under shade only to 16.0% with cooling using sprinklers and forced ventilation, representing a relative increase of approximately 158% [68]. Furthermore, cooling time, as short as two hours before and after insemination, increase significantly pregnancy rates in cattle up to twice, from 23% under heat stress to 56% with cooling treatment [68]. In facilities with these cooling systems, embryo transfer recipients maintain pregnancy rates around 34%, compared to 15% for those under heat stress without cooling [69].

### Genetic approaches

Selective breeding strategies enhance thermotolerance in cattle by targeting genes associated with natural heat resilience. The *ATP1A1* gene, which is associated with cell membrane stability, has been linked to heat tolerance in cattle. Cattle carrying the C allele at the C2789A locus, particularly those with the CC genotype, maintain rectal temperatures approximately 0.5°C lower during heat waves and show a 15% improvement in conception rates [70]. Similarly, the *SOD1* gene, which reduces oxidative stress, contributes to a 10%–12% increase in conception rates during summer in cattle selected for high *SOD1* expression [71]. Additionally, the *HSPA1L* deletion boosts cellular resilience by amplifying heat shock protein expression, which improves cell survival under heat stress. Embryos from bulls with the *HSPA1L* mutation demonstrated higher blastocyst formation rates, indicating improved viability under elevated temperatures [72]. These genetic adaptations provide a foundation for developing heat-tolerant breeds through selective breeding.

Studies have demonstrated that conception rates for Brown Swiss, Jersey, and Holstein in Florida dropped from 52% to 32% as temperatures rose from 23.9°C to 32.2°C. In contrast, Brahman, which possess innate thermoregulatory strengths, maintain stable oocyte quality and embryo development across seasons. This highlights the potential for incorporating *Bos indicus* genetics into *Bos taurus* breeds to enhance heat resilience and sustain reproductive efficiency in challenging climates [28,73]. Gene editing further contributes to thermotolerance improvements through specific mutations. The SLICK1 mutation by gene editing has shown substantial gains in heat tolerance. Slick Holsteins, with their shorter, less insulating coats, maintained lower rectal temperatures, often staying below 39.5°C in summer conditions where ambient temperatures frequently exceeded 32°C. This mutation effectively mitigates the impact of high temperatures on cattle by enhancing thermoregulation [74]. In 2022, the U.S. FDA approved SLICK cattle, which were CRISPR-edited for enhanced heat resilience, and confirmed their safety based on genomic data [75]. Despite such advances, selective breeding remains a more practical and broadly accepted strategy within existing regulatory frameworks.

### Supportive treatments

#### Pharmacological strategies

Supportive treatments to enhance reproductive performance in heat-stressed cattle involve both targeted nutritional supplementation and hormonal interventions [76,77]. Among these, melatonin is widely used to mitigate oxidative stress in oocytes and embryos [78,79]. Melatonin lowers oxidative stress by reducing ROS by 50% and increasing glutathione peroxidase activity by 30%. It also inhibits the NF-κB inflammatory pathway, resulting in a 40% reduction in pro-inflammatory cytokines such as TNF-α. By stabilizing mitochondrial ATP production, melatonin reduces mitochondrial ROS formation by 25%, helping to maintain energy balance and cellular integrity. These protective effects lead to tangible reproductive benefits, including a 10%–15% reduction in open days and up to a 30% decrease in early pregnancy losses, particularly when administered during the dry-off period [80].

Chromium supplementation enhances insulin sensitivity by increasing insulin receptor phosphorylation and promoting glucose transporter 4 (GLUT4) translocation to cell membranes, which facilitates glucose uptake and reduces blood glucose levels by approximately 15%–20% [81]. This action alleviates hyperinsulinemia, a common issue under heat stress, thereby protecting ovarian follicles from metabolic stress [82]. Additionally, chromium reduces ROS levels by up to 25% and lowers pro-inflammatory cytokines like TNF-α and interleukin-6 (IL-6) by 30%–35%, mitigating inflammation and maintaining a healthy follicular environment. In trials, these effects collectively improved early pregnancy rates by 20%–25% in heat-stressed cattle, promoting metabolic and reproductive stability [76].

Methionine, a key methyl donor in the DNA methylation cycle, supports embryo development by serving as a precursor for S-adenosylmethionine, facilitating DNA and histone methylation crucial for epigenetic regulation and proper gene expression [83]. This process enhances embryo survival and quality, with studies reporting up to a 20% improvement in pregnancy rates. Methionine also acts as a precursor for glutathione, increasing antioxidant enzyme activity by 20%–30% and mitigating oxidative damage during heat stress. Furthermore, methionine supplementation shortens estrous cycles, enhancing reproductive efficiency [41,84].

Similarly, betaine contributes to reproductive performance through its dual role as a methyl donor and an osmolyte. By supporting the methionine cycle, betaine facilitates epigenetic regulation and enhances embryo viability [85]. In its osmoprotective function, betaine stabilizes intracellular osmotic balance, preserving protein structure and cellular hydration under heat stress. Therefore, betaine supplementation has been shown to improve conception rates by 10%–15%, reduce calving complications, and shorten the interval from calving to first estrus, thereby strengthening reproductive resilience in heat-stressed cattle [76].

Antioxidant supplementation has emerged as a promising pharmacological strategy to mitigate heat-induced impairment of sperm quality in bulls. Supplementation with melatonin, vitamin E, and selenium has been shown to counteract these detrimental effects by scavenging ROS and restoring redox balance [86,87]. Melatonin improves motility and overall semen parameters following oxidative or thermal insults [88], while selenium enhances mitochondrial activity and plasma membrane integrity, leading to improved sperm viability [87]. Vitamin E stabilizes sperm membranes and reduces DNA fragmentation, thereby supporting normal spermatogenesis [86,89]. Experimental and clinical studies demonstrate that antioxidant supplementation can restore sperm motility, viability, and hormone profiles during or after heat exposure [90]. These findings indicate that targeted antioxidant supplementation is an evidence-based approach to accelerate recovery from heat-induced reproductive impairment in bulls.

#### Hormonal interventions

Hormonal interventions are critical for managing the hormonal disruptions in estrous and ovulation cycles caused by heat stress, offering targeted strategies to enhance reproductive performance and support uterine health. Gonadotropin-releasing hormone (GnRH) administered at AI stimulates the pituitary to release LH and follicle-stimulating hormone (FSH), which induce ovulation and synchronize follicular waves. This synchronization has been shown to improve conception rates by 10%–15% in heat-stressed cattle and can also enhance uterine conditions by promoting timely follicular turnover [91]. Progesterone-based protocols, particularly when combined with equine chorionic gonadotropin (eCG) or human chorionic gonadotropin (hCG), are effective for resolving anestrus and promoting synchronized ovulation. Progesterone creates a stable uterine environment by maintaining endometrial readiness and minimizing inflammatory responses [92]. Upon removal before AI, progesterone triggers a controlled LH surge, resulting in synchronized ovulation. eCG acts similarly to FSH, enhancing follicular development to produce larger, more viable follicles, which are critical for successful conception [93]. Meanwhile, hCG acts like LH to extend corpus luteum function, thereby sustaining progesterone levels essential for early pregnancy maintenance and a supportive uterine environment for embryo development [94]. In trials, combining progesterone with eCG or hCG has demonstrated a significant increase in pregnancy rates, with improvements of up to 20%–25% in heat-stressed cattle compared to standard AI protocols [95]. These hormonal strategies also reduce the interval from calving to conception, enhancing overall reproductive efficiency under heat stress conditions. By directly addressing hormonal and uterine disruptions, these combined protocols offer a comprehensive approach to overcoming fertility challenges associated with heat stress, thereby promoting both embryo survival and uterine health essential for maintaining pregnancies [96].

#### Limitations and unmet needs

While current supportive treatments provide meaningful benefits by reducing oxidative stress, controlling inflammation, and regulating estrous cycles, their therapeutic effects remain limited and most interventions focus on symptomatic management and cycle control, offering only partial protection against the multifaceted cellular and metabolic disruptions induced by heat stress. Moreover, hormonal protocols, although effective, increase treatment complexity and labor costs, especially in large-scale herd settings. These limitations highlight an unmet need for next-generation strategies grounded in a comprehensive understanding of heat stress pathophysiology. To improve both efficacy and field applicability, future approaches should target the underlying cellular dysfunctions, while ensuring safety and sustainability across diverse production systems.

## FUTURE PROSPECTS AND RESEARCH DIRECTIONS

**Figure 1:**
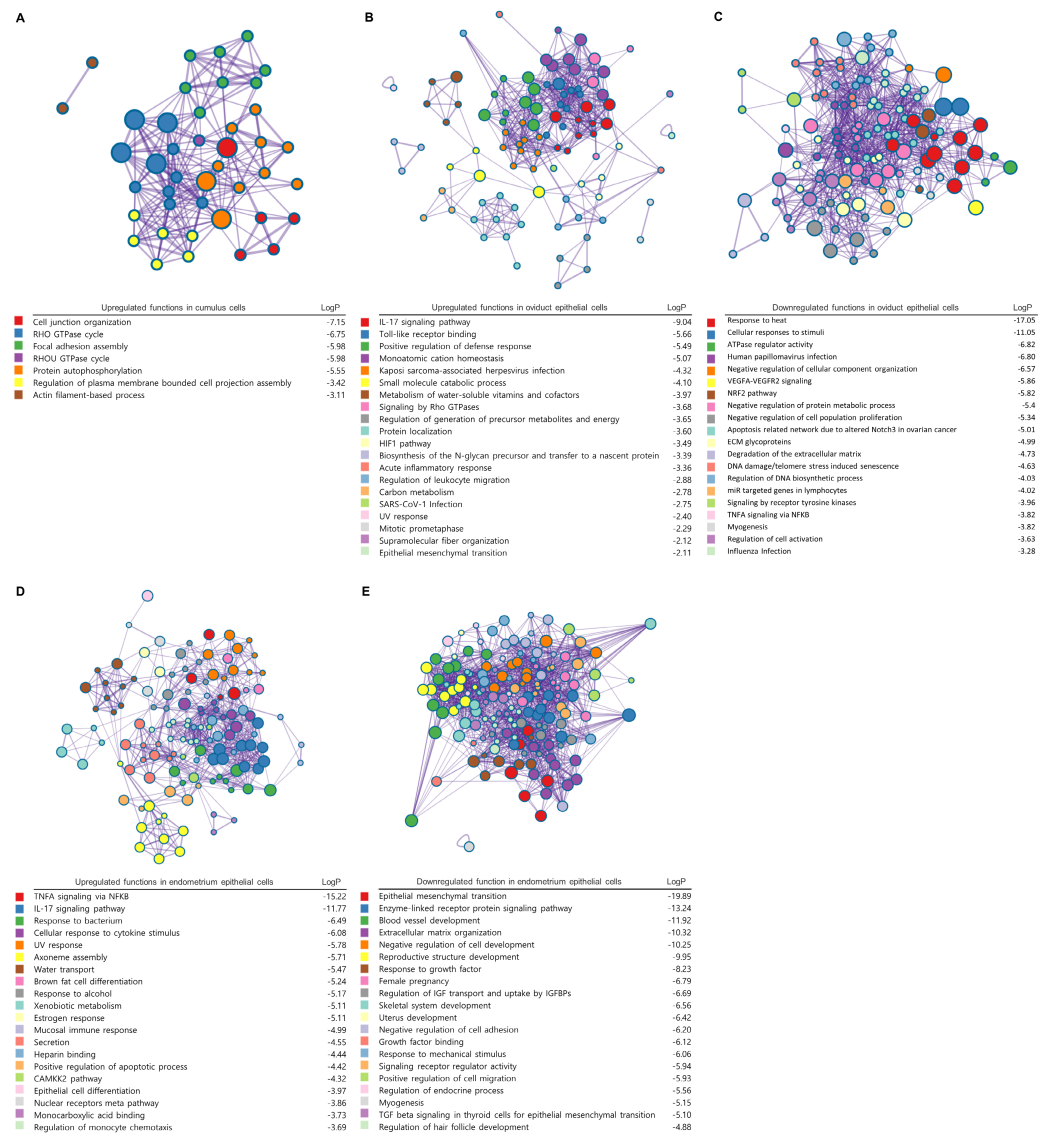
Functional enrichment analysis of heat stress-induced transcriptomic changes in reproductive cells of female cattle. Heat stress-induced transcriptional and functional responses across the female reproductive tract were analyzed using an integrated re-analysis of publicly available multi-omics datasets (GSE66057, GSE221895, GSE235170, and GSE235171) along with published data [97–101]. Functional enrichment analyses were performed on differentially expressed genes (DEGs) identified in each cell type under heat stress conditions. (A) Upregulated functions in cumulus cells following heat stress, highlighting pathway enrichment related to cytoskeletal remodeling (actin filament-based processes, cell projection organization), integrin-mediated adhesion, and ephrin signaling, including key GTPase cycles (RHOA, RAC1, CDC42) that may impact cumulus-oocyte complex (COC) integrity. (B) Upregulated functions in oviductal epithelial cells under heat stress, showing prominent activation of innate immune responses, such as neutrophil chemotaxis, defense response, IL-17, and TNF signaling pathways. (C) Downregulated functions in oviductal epithelial cells upon heat stress, indicating broad suppression of molecular chaperones (HSP70, HSP90, α-crystallin) and their upstream regulators (HSF1, TP53, NRF2), as well as reduced steroid hormone receptor signaling, with potential impairment of protein homeostasis and epithelial integrity. (D) Upregulated functions in endometrial epithelial cells exposed to heat stress, demonstrating pronounced activation of inflammation (TNF, IL-17 signaling), cell death pathways, and immune activation, ultimately compromising epithelial homeostasis. (E) Downregulated functions in endometrial epithelial cells following heat stress, including pathways related to angiogenesis, cellular differentiation, extracellular matrix remodeling, and hormone signaling, which are associated with impaired endometrial receptivity and increased risk of implantation failure. Each node represents an enriched biological subfunction defined by DEGs, and each edge indicates a functional connection based on shared genes between subfunctions. Network was generated by Metascape (https://metascape.org/).

Building on this need, recent advances in multi-omics technologies have opened new avenues to dissect the molecular pathogenesis of heat stress at unprecedented resolution. To demonstrate the utility of such an approach, heat stress-induced transcriptional and functional responses across the female reproductive tract were analyzed using an integrated re-analysis of publicly available multi-omics datasets (GSE66057, GSE221895, GSE235170, and GSE235171) along with published data [96–100]. Considering the particular susceptibility of oocyte maturation, early embryonic development, and implantation to heat stress, the re-analysis focused on cumulus cells, oviductal epithelium, and endometrial epithelium. Systems-level comparisons reveal both conserved cellular responses and cell type-specific vulnerabilities, including disruption of chaperone-mediated protein folding, immune dysregulation, impaired steroid signaling, and cytoskeletal destabilization. In cumulus cells, heat stress enhances Rho GTPase and integrin signaling, altering cytoskeletal dynamics and potentially weakening cumulus–oocyte interactions. Oviductal epithelial cells exhibit marked activation of pro-inflammatory and apoptotic pathways, along with suppression of chaperone systems and hormone receptor signaling, which may disturb the fertilization environment. In endometrial epithelial cells, elevated immune infiltration and cellular stress are accompanied by downregulation of developmental and structural pathways essential for uterine receptivity (Figure 1). These findings provide critical molecular entry points for precision intervention. Future research should focus on clarifying the cellular and molecular pathogenesis of heat stress and identifying actionable pharmacological targets. Tools such as multi-omics integration, spatial profiling, and network-based modeling offer valuable means to achieve these goals, but the ultimate objective lies in translating mechanistic insights into practical solutions. This includes discovering and validating novel bioactive compounds that can restore cellular homeostasis, modulate excessive immune responses, and preserve the reproductive microenvironment under thermal stress. Advancing such strategies will be essential for developing next-generation interventions that are not only mechanistically rational but also safe, effective, and applicable in real-world cattle reproduction systems. At the same time, approaches such as multi-omics-guided drug discovery face practical challenges related to cost and regulatory hurdles, underscoring the need for continued translational research and supportive policies.

## Figures and Tables

**Table 1:** Physiological responses of cattle to heat stress

Environment	Temperature (°C)	Humidity (%)	Rectal temp (°C)	Respiration (breaths/min)	Heart rate (beats/min)	Surface temp (°C)
Natural	10–22	14–50	38.4–39.1	25–50	69–82	Not reported
Natural	28–41	15–78	38.7–40.1	43–102	71–83	Not reported
Natural (with cooling)	28–42	Not reported	38.6–40.0	42–91	78–83	Not reported
Chamber	17–21	55–65	38.3–38.8	~47	Not reported	31.3–34.1
Chamber	Up to ~39	55–67	38.9–40.2	105	Not reported	35.4–38.2

Data from Gwazdauskas [5] and Rhoads et al [6].

## Data Availability

Upon reasonable request, the datasets of this study can be available from the corresponding author.
